# Ecology, seasonality and host preferences of Austrian *Phlebotomus* (*Transphlebotomus*) *mascittii* Grassi, 1908, populations

**DOI:** 10.1186/s13071-021-04787-2

**Published:** 2021-05-29

**Authors:** Edwin Kniha, Markus Milchram, Vít Dvořák, Petr Halada, Adelheid G. Obwaller, Wolfgang Poeppl, Gerhard Mooseder, Petr Volf, Julia Walochnik

**Affiliations:** 1 Institute of Specific Prophylaxis and Tropical Medicine, Center for Pathophysiology, Infectiology and Immunology, Medical University of Vienna, Vienna, Austria; 2 Institute of Zoology, Department of Integrative Biology and Biodiversity Research, University of Natural Resources and Life Sciences, Vienna, Austria; 3 Department of Parasitology, Faculty of Science, Charles University, Prague, Czech Republic; 4 BioCeV, Institute of Microbiology of the Czech Academy of Sciences, Vestec, Czech Republic; 5 Division of Science, Research and Development, Federal Ministry of Defence, Vienna, Austria; 6 Department of Dermatology and Tropical Medicine, Military Medical Cluster East, Austrian Armed Forces, Vienna, Austria

**Keywords:** Phlebotomine sand fly, Central Europe, Climate, Blood meal, MALDI-TOF

## Abstract

**Background:**

Sand flies are principal vectors of the protozoan parasites *Leishmania* spp. and are widely distributed in all warmer regions of the world, including the Mediterranean parts of Europe. In Central European countries, the sand fly fauna is still under investigation. *Phlebotomus mascittii*, a suspected but unproven vector of *Leishmania infantum*, is regarded as the most widely distributed species in Europe. However, many aspects of its biology and ecology remain poorly known. The aim of this study was to provide new data on the biology and ecology of *Ph. mascittii* in Austria to better understand its current distribution and potential dispersal.

**Methods:**

Sand flies were collected by CDC light traps at four localities in Austria for 11 (2018) and 15 weeks (2019) during the active sand fly season. Climatic parameters (temperature, relative humidity, barometric pressure and wind speed) were retrospectively obtained for the trapping periods. Sand flies were identified by a combined approach (morphology, DNA barcoding, MALDI-TOF protein profiling), and blood meals of engorged females were analysed by DNA sequencing and MALDI-TOF mass spectrometry.

**Results:**

In total, 450 individuals of *Ph. mascittii* were caught. Activity was observed to start at the beginning of June and end at the end of August with peaks in mid-July at three locations and early August at one location. Increased activity was associated with relatively high temperatures and humidity. Also, more individuals were caught on nights with low barometric pressure. Analysis of five identified blood meals revealed chicken (*Gallus gallus*) and equine (*Equus* spp.) hosts. Sand fly abundance was generally associated with availability of hosts.

**Conclusion:**

This study reports unexpectedly high numbers of *Ph. mascittii* at selected Austrian localities and provides the first detailed analysis of its ecology to date. Temperature and humidity were shown to be good predictors for sand fly activity. Blood meal analyses support the assumption that *Ph. mascittii* feeds on mammals as well as birds. The study significantly contributes to understanding the ecology of this sand fly species in Central Europe and facilitates prospective entomological surveys.

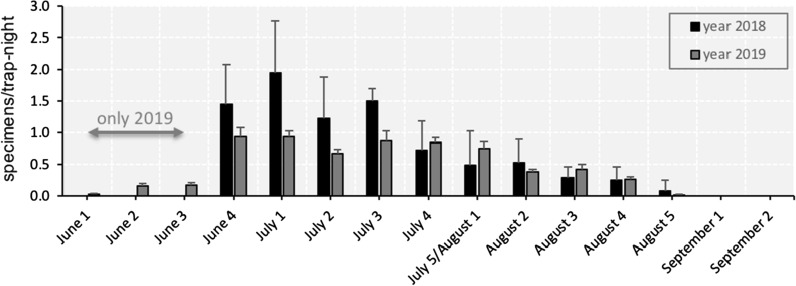

**Supplementary Information:**

The online version contains supplementary material available at 10.1186/s13071-021-04787-2.

## Introduction

Phlebotomine sand flies (Diptera: Psychodidae: Phlebotominae) are small hematophageous insects. During a blood meal, females can transmit various pathogens, including the protozoan parasites *Leishmania* spp., the α-proteobacterium *Bartonella bacilliformis* and various arthropod-borne viruses [1].

The occurrence of sand flies in Central Europe, north of the Alps, had long been questioned, but then *Phlebotomus mascittii* Grassi, 1908, was first found in Germany in 1999 [2]. In the following years, *Ph. mascittii* was also found in five federal states of Austria and in Western Slovakia, close to the Austrian border [3–6]. *Ph. mascittii* is understood to have a wide geographical range with collections reported from Switzerland, France, Belgium and Germany. With records as far as 50° North, it is the northernmost occurring sand fly species in Europe [7–9]. It is also known to occur in western parts of the Mediterranean [10, 11], while reports from eastern parts of the Mediterranean [12] are likely to represent the recently described species *Phlebotomus killicki* Dvořák, Votýpka & Volf, 2015 [13].

Temperature is considered the most critical factor for sand fly dispersal and activity. Humidity also constitutes a crucial factor, as a moist substrate is required for egg and larval development [14]. In Central Europe, sand flies diapause during cold winter months, and while sand fly activity usually stretches from spring to autumn in Mediterranean regions, sand flies in more northern countries such as Germany, Austria and Slovakia are mainly active in the summer [2, 4, 6]. Winter activity has only been reported from trappings in a tunnel on the island of Corsica, where a cave-like blocked railway tunnel exhibits stable climatic conditions year-round; temperatures > 15 °C were measured in February inside the tunnel, thereby providing suitable conditions for sand fly activity during winter time [15].

*Phlebotomus mascittii* is a suspected but unproven vector for *Leishmania infantum* based on circumstantial evidence and close phylogenetic relationship to the subgenera *Larroussius* and *Adlerius*, which include important vector species of *L. infantum* [16]. Moreover, presumably autochthonous leishmaniasis cases have been reported from Austria [17] and Germany [18], and *L. infantum* DNA was detected in an unfed Austrian *Ph. mascittii* specimen and in a specimen caught on the Italian island of Montecristo [19, 20].

While *Ph. mascittii* is the most commonly found sand fly species in Central Europe, knowledge on its distribution, ecology and activity is still scarce. To fill this gap, we performed a detailed study during the summer months of 2018 and 2019, assessing sand fly activity and associated climatic and ecological factors at various locations in Austria.

## Materials and methods

### Study area and environment

**Fig. 1:**
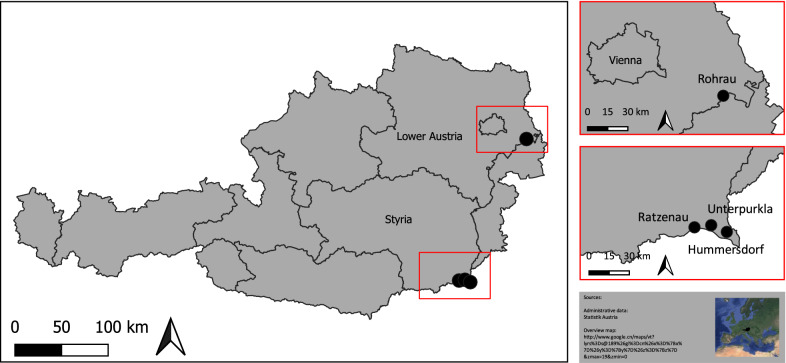
Map of trapping sites

The survey was conducted in two different federal states of Austria at four different trapping sites, namely Rohrau (Ro) in Lower Austria and Ratzenau (Ra), Unterpurkla (Up) and Hummersdorf (Hu) in Styria (Fig. 1). Rohrau is located at the border of the two federal states Lower Austria and Burgenland in Eastern Austria. The mean January and July temperatures (20-year mean, 1999–2019) are − 1.1 and 20.0 °C, respectively, and the total annual precipitation is 629 mm. Ratzenau, Unterpurkla and Hummersdorf are located in the southern part of Styria along the Slovenian border. In this region, mean January and July temperatures are − 1.4 and 19.8 °C, respectively, and the total annual precipitation is 873 mm. Both trapping areas belong to the warmest parts of Austria and are classified as Cfb (*C* = temperate, *f* = no dry season, *b* = warm summer) according to Köppen and Geiger.

The trapping sites exhibit optimal climatic conditions and the presence of sand flies was proven in previous studies [4, 5]. While in Rohrau the only available large building was chosen for sampling, two buildings at each trapping site were sampled in Ratzenau, Unterpurkla and Hummersdorf (Table 1).

**Table 1:** Long-term trapping sites in Austria

Site	Latitude, longitude	Altitude (m.a.s.l.)	Traps	Site description	Potential host^a^
Rohrau (Ro)	48.0657, 16.8593	148 m	Ro1, Ro2, Ro3	Old large barn on private property with natural floor	Dog always present, humans
Ratzenau (Ra)	46.7266, 15.8243	233 m	Ra1, Ra2	Old garage with trash, old barn with natural floor	Dog close, rodents, bats, humans
Unterpurkla (Up)	46.7319, 15.9062	222 m	Up1, Up2	Old barn with wood, large old garage with hay	Cats, rodents, humans, chicken
Hummersdorf (Hu)	46.7076, 15.9812	209 m	Hu1, Hu2	Old barn with trash, poultry barn	Dog close, rodents, humans close, chicken

^a^Potential host within 50 m radius

### Sand fly trapping

Sand fly trapping was conducted using battery-operated CDC miniature light traps with fine gossamer collection bags (model #512, John W. Hock Co., Gainesville, FL, USA) from 28 June to 31 August 2018 in Rohrau and from 28 June to 7 September 2018 in Ratzenau, Unterpurkla and Hummersdorf. Based on observations and trapping numbers in 2018, trappings in 2019 were scheduled earlier, started on 4 June and ran until 13 September at all locations.

Trappings were carried out every week from Monday to Friday, resulting in 4 consecutive nights sampled per week with exceptions of bank holidays. In 2019, the trapping site in Rohrau was only sampled from Monday to Thursday, resulting in 3 consecutive nights sampled per week. The trapping approach resulted in 104 total nights trapped (801 trap-nights = nights*traps), of which 46 nights (369 trap-nights) were sampled in 2018 and 58 (Styria) and 45 (Lower Austria) nights (432 trap-nights) in 2019.

Collection bags were changed every morning after sunrise. The contents were carefully transferred to Petri dishes labelled with date, location and trap number and stored at − 20 °C until further inspection. Sand fly specimens were sorted using a stereomicroscope Wild Heerbrug M8 (Wild Heerbrug, Sankt Gallen, Switzerland) with low magnification, pre-sorted by sex and transferred to 70% ethanol for preservation.

### Morphological identification

Head and genitalia were dissected and slide-mounted in CMCP-10 mounting medium (Polysciences, Germany). Identification was based on published morphological keys and descriptions of male genitalia, female spermatheca and pharyngeal armature [8, 21].

### Molecular identification

DNA was isolated from the remaining body parts with a QIAamp® DNA Mini Kit 250 (Qiagen, Hilden, Germany). PCR amplification of a 658-basepairs (bp) fragment of the cytochrome c oxidase subunit I (*coxI*) gene was performed using the primers LCO-1490/CoxUniEr following the protocol of Kniha et al. [22].

PCR was performed with an Eppendorf Mastercycler (Eppendorf AG, Hamburg, Germany). Bands were analysed with a Gel Doc™ XR + Imager (Bio-Rad Laboratories Inc., California, USA), cut out from the gel and purified with an Illustra™ GFX™ PCR DNA and Gel Purification Kit (GE Healthcare, Buckinghamshire, UK). Sequencing was performed with a Thermo Fisher Scientific SeqStudio (Thermo Fisher Scientific, Massachusetts, USA). Obtained sequences from both strands were aligned with ClustalX 2.1, edited with GeneDoc 2.7.0 and consensus sequences were blasted in the NCBI sequence database (GenBank) and compared to reference sequences.

MALDI-TOF protein profiling was done as previously described [23, 24]. The protein extracts from thoraces of chosen specimens were mixed with a sinapinic acid matrix and mass spectra were acquired with an Ultraflex III MALDI-TOF spectrometer (Bruker Daltonics, Bremen, Germany). The spectra were visualized by FlexAnalysis 3.4 software, processed by MALDI Biotyper 3.1 and compared with an in-house reference database.

### Blood meal analysis by DNA sequencing and MALDI-TOF mass spectrometry

PCR was performed in volumes of 50 μl as described above. Primer pairs PNOC-F/PNOC-R and PCR cycles were performed as described by Haouas et al. [25] and sequencing was performed as described above. Obtained sequences were submitted to GenBank. A male *Ph. mascittii* specimen and filtered H_2_O were used as negative controls.

MALDI-TOF peptide mass mapping analysis of host-specific hemoglobin peptides was performed according to a protocol by Hlavackova et al. [26]. Blood from engorged abdomens was digested using trypsin (Promega) and the resulting peptides were mixed with an α-cyano-4-hydroxycinnamic acid matrix (Bruker Daltonics). Peptide mass maps were acquired with an Ultraflex III MALDI-TOF instrument (Bruker Daltonics) and at least two peptides per female were selected for MS/MS sequencing. MS/MS spectra were searched against the SwissProt 2019_05 database subset of vertebrate proteins using an in-house MASCOT search engine (Matrix Science).

### Meteorological data

Climatic data, including temperature, relative humidity, air pressure and wind speed, were obtained from the Central Institute for Meteorology and Geodynamics (ZAMG). Daily, weekly and monthly means and standard deviations were calculated for daytime (sunrise to sunset) and nighttime (sunset to sunrise).

### Statistical analysis

All data were analysed using R 3.6.2 [27]. To compare the trapping success between different years, sexes and reproduction status, we applied tests of proportions and Kruskal-Wallis tests.

To investigate the influences of weather variables, we fitted negative binomial zero-inflated generalized linear mixed models (ZIGLMM) with a log link function using the glmmTMB package [28] with trap-night and sampling location at each trapping site as random factors. We fitted captures per trap-night as response variable and tested mean daily humidity, mean nightly humidity, mean nightly temperature, mean nightly air pressure (all continuous) and trapping sites (categorical with four levels) as fixed factors:

$${\text{log (C}}_{{{\text{ij}}}} {\text{) = Hum\_n}}_{{{\text{ij}}}}^{{2}} {\text{ + Hum\_d}}_{{{\text{ij}}}} {\text{ + Temp\_n}}_{{{\text{ij}}}} {\text{ + Press\_n}}_{{{\text{ij}}}} {\text{ + Site}}_{{\text{j}}} {\text{ + Temp}}_{{{\text{ij}}}} \, \times {\text{ Hum\_n}}_{{{\text{ij}}}} {\text{ + Loc}}_{{\text{i}}} {\text{ + Date}}_{{\text{j}}}$$

where *C* is the *j*th number of captured individuals at location *i*.

Since both mean nightly temperature/mean daily temperature and mean nightly air pressure/mean daily air pressure were correlated (Pearson’s correlation coefficient > 0.6), we included only nightly data of those variables as fixed factors.

We built models by backward elimination and chose the one with the lowest Akaike information criterion [29] as the best fitting model. Then, we used the DHARMa package [30] for model validation and ggplot2 [31] and ggeffects [32] for the visualisation of the model output.

## Results

### Sand fly identification

Overall, 450 specimens were caught, which were all identified as *Phlebotomus mascittii* by morphological characters. Further confirmation was obtained by sequencing a *coxI* gene region of one male and one female of each of the four locations (GenBank accession: MW741695.1–MW741702.1) and MALDI-TOF protein profiling of chosen specimens from different trapping localities, which were hardly or not identifiable to the species level by morphology. Obtained *coxI* sequences showed 100% identity when compared to reference sequences from GenBank (MN003381.1, KX869078.1). MALDI-TOF protein profiling of 21 specimens collected from four localities (Hummersdorf 10 specimens, Unterpurkla 4 specimens, Ratzenau 6 specimens, Rohrau 1 specimen) provided species-specific protein spectra that confirmed species identification as *Ph. mascittii*.

### Trapping numbers

Out of 450 caught specimens, 271 (60.2%) were trapped in 2018 and 179 (39.8%) in 2019, accounting for 0.7 and 0.4 caught specimens/trap-night in 2018 and 2019, respectively (Table 2). Of 801 total trap-nights, significantly fewer trap-nights were successful than unsuccessful (213 vs. 588; *P* < 0.001), and this was observed for both years, 2018 (113 vs. 256; *P* < 0.001) and 2019 (100 vs. 332; *P* < 0.001), independently.

**Table 2:** Number of trapped *Ph. mascittii* by year, sex, feeding status and site

Category	Year	Rohrau	Ratzenau	Unterpurkla	Hummersdorf	Total
Male (%)	2018	4 (7.0%)	3 (5.3%)	7 (12.3%)	43 (75.4%)	57 (21.0%)
	2019	5 (25.0%)	2 (10.0%)	2 (10.0%)	11 (55.0%)	20 (11.2%)
	Total	9 (11.7%)	5 (6.5%)	9 (11.7%)	54 (70.1%)	77 (17.1%)^a^
Female (%)	2018	10 (4.7%)	48 (22.4%)	73 (34.1%)	83 (38.8%)	214 (79.0%)
	2019	18 (11.3%)	18 (11.3%)	76 (47.8%)	47 (29.6%)	159 (88.8%)
	Total	28 (7.5%)	66 (17.7%)	149 (39.9%)	130 (34.9%)	373 (82.9%)^a^
Engorged (%)	2018	2 (15.4%)	3 (23.1%)	3 (23.1%)	5 (38.5%)	13 (86.7%)
	2019	0 (0.0%)	0 (0.0%)	0 (0.0%)	2 (100.0%)	2 (13.3%)
	Total	2 (13.3%)	3 (20.0%)	3 (20.0%)	7 (46.7%)	15 (100.0%)
Gravid (%)	2018	4 (25.0%)	3 (18.8%)	2 (12.5%)	7 (43.8%)	16 (80.0%)
	2019	3 (75.0%)	1 (25.0%)	0 (0.0%)	0 (0.0%)	4 (20.0%)
	Total	7 (35.0%)	4 (20.0%)	2 (10.0%)	7 (35.0%)	20 (100.0%)
Total (%)	2018	14 (5.2%)	51 (18.8%)	80 (29.5%)	126 (46.5%)	271 (60.2%)
	2019	23 (12.8%)	20 (11.2%)	78 (43.6%)	58 (32.4%)	179 (39.8%)
	Total	37 (8.2%)	71 (15.8%)	158 (35.1%)	184 (40.9%)	450 (100.0%)

^a^Percentage of all caught specimens

In total, 77 (17.1%) specimens were males and 373 (82.9%) were females, of which 15 (4.0%) were engorged and 20 (5.4%) were gravid (Table 2). In both years, significantly more females were caught than males (2018: 214 vs. 57; *P* < 0.001; 2019: 159 vs. 20; *P* < 0.001).

The overall male/female ratio was 1/4.8, being split to 1/3.8 in 2018 and 1/8.0 in 2019. While high male/female ratios were observed in Hummersdorf (1/2.4) and Rohrau (1/3.1), low male/female ratios were observed in Unterpurkla (1/13.2) and Ratzenau (1/16.6).

The number of collected specimens varied between trapping sites. The highest capture rate was observed in Hummersdorf (184, 0.9 specimens/trap-night) and a slightly lower rate in Unterpurkla (158, 0.8 specimens/trap-night). Trapping success was clearly lower in Ratzenau (71, 0.4 specimens/trap-night) and Rohrau (37, 0.2 specimens/trap-night) (Table 2).

The overall highest number was caught with trap Up2 (109/450; 24.2%) followed by Hu1 (108/450; 24.0%), together accounting for almost half of all caught specimens (Additional file 1: Table S1).

### Seasonal abundance

**Fig. 2:**
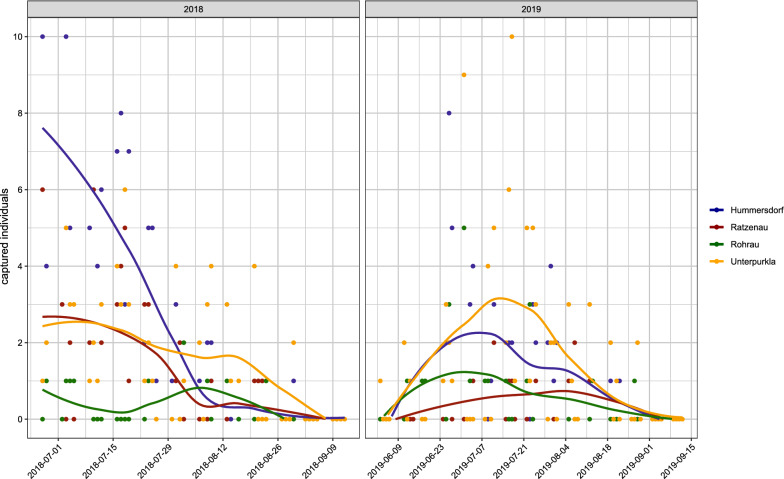
Seasonal sand fly activity by year and location

In 2018, sand fly activity was recorded from 28 June to 23 August in Styria and from 29 June to 31 August in Lower Austria (Fig. 2, Additional file 2: Table S2). However, as first records were obtained already in the first trap night, we assume that activity had started before monitoring in 2018; thus, no further calculations on seasonal abundance were made.

In 2019, the beginning of sand fly activity was observed on 4 June and 13 June in Styria and Lower Austria, respectively. Again, a single specimen was trapped in the first trap night, but only in Unterpurkla. Activity ended between 20 and 29 August in Styria and on 28 August in Lower Austria (Fig. 2, Additional file 2: Table S2). The mean sand fly activity period was 70 (SD: 14) days, with the shortest period in Ratzenau (54 days) and longest in Unterpurkla (86 days) and Rohrau (76 days).

In 2019, sand fly abundance showed a monomodal curve at all four locations. In Unterpurkla, Hummersdorf and Rohrau sand fly activity peaked in early July, while in Ratzenau sand fly activity peaked at the beginning of August (Fig. 2). Overall, most specimens were trapped in the last week of June (29, 16.2%) and the first week of July (29, 16.2%). While male sand fly abundance peaked in the last week of June (7, 35.0%), female sand fly abundance peaked in the third week of July (27, 17.0%).

### Sand fly activity and climatic conditions

**Fig. 3:**
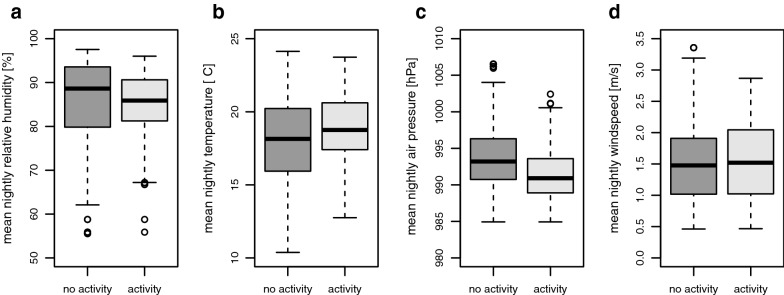
Boxplots of sand fly trapping success according to climatic parameters relative humidity (**a**), temperature (**b**), air pressure (**c**) and windspeed (**d**)

Sand flies were active between 56 and 96% mean nightly relative humidity and 12.8 °C and 23.7 °C mean nightly temperature (Fig. 3). Earliest sand fly activity was noticed only after the mean temperature and the minimum temperature did not fall below 15 °C and 10 °C for 5 consecutive days, respectively. Sand fly activity was observed between 985 and 1002 hPa mean nightly air pressure and 0.5 and 2.9 m/s mean nightly wind speed (Fig. 3).

**Fig. 4:**
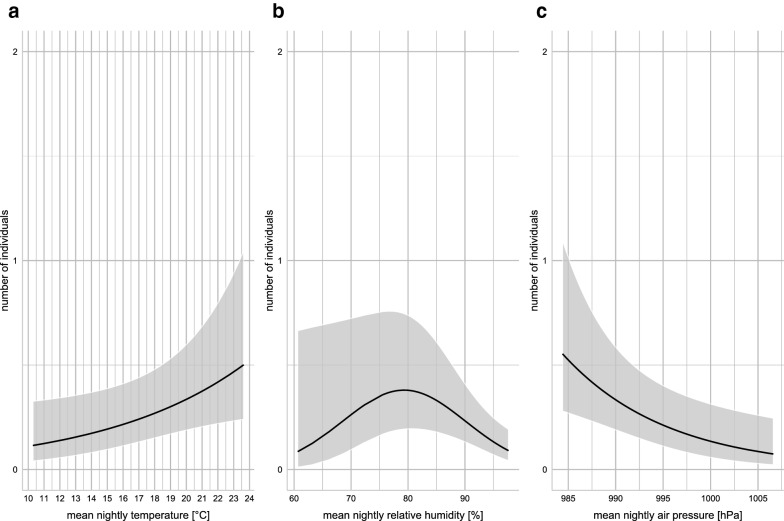
Predictions of the sand fly activity based on a zero-inflated generalized linear mixed model for temperature (**a**), humidity (**b**) and air pressure (**c**)

Model validation did not reveal any problems. In our best model, mean nightly air humidity had a unimodal effect on the trapping success (Fig. 4b, Additional file 3: Figure S1). Higher mean nightly temperature and an interaction term of mean nightly temperature and mean nightly humidity influenced the trapping success positively (Fig. 4a, Additional file 4: Figure S2), while higher mean nightly air pressure decreased the number of captured individuals per night (Fig. 4c, Additional file 5: Figure S3). The numerical output of the best ZIGLMM is shown in Table 3.

**Table 3:** Estimates, standard errors, *z* values and *P* values for the best negative binomial ZIGLMM

	Estimate	Std. error	*z* value	*P* value
(Intercept)	− 0.440	0.276	− 1.593	0.111
SiteRatzenau	− 0.800	0.238	− 2.441	< 0.05
SiteRohrau	0.864	0.445	− 1.941	0.052
SiteUnterpurkla	− 0.090	0.312	− 0.287	0.774
Hum_n	− 0.509	0.162	− 3.141	< 0.01
Hum_n^2^	− 0.267	0.108	− 2.471	< 0.05
Temp_n	0.261	0.142	1.837	0.066
Press_n	− 0.399	0.146	− 2.726	< 0.01
Temp_n:Hum_n	0.286	0.125	2.297	< 0.05

The variance of the random factor “location” *σ*_Loc_ is 0.07 and of the random factor “trap-night” *σ*_Date_ is 0.750. The best model does not include mean daily humidity (Hum_d)

### Blood meal analysis

All 15 engorged females were subjected to PCR as well as MALDI-TOF analysis, and five blood meals were successfully identified. MALDI-TOF peptide mass mapping detected hemoglobin fragments of chicken (*Gallus gallus*) in one specimen from Unterpurkla and in two specimens from Hummersdorf. PCR and sequencing revealed equine DNA (*Equus* spp.) in one specimen from Ratzenau (GenBank accession: MT712271.1) and in one specimen from Unterpurkla (GenBank accession: MT712272.1). The resulting sequences originating from specimens from Ratzenau and Unterpurkla with a length of 287 bp showed 98.2 to 100% and 97.9 to 99.7% identity, respectively, with reference sequences of *Equus asinus* as well as *E. caballus* from GenBank (Table 4).

**Table 4:** Blood meal analysis of *Ph. mascittii* by MALDI-TOF and sequencing

Site	Method	Identified	Blood meal	Identity	Reference
Ra	MALDI-TOF	–	–	–	–
	PCR	1	*Equus spp.*	282/282 bp (100%) 281/282 bp (99.7%)	*Equus asinus* (KM521860.1) *Equus caballus* (AY011855.1)
Up	MALDI-TOF	1	*Gallus gallus*	–	–
	PCR	1	*Equus spp.*	277/282 bp (98.2%) 276/282 bp (97.9%)	*Equus asinus* (KM521860.1) *Equus caballus* (AY011855.1)
Hu	MALDI-TOF	2	*Gallus gallus*	–	–
	PCR	–	–	–	–

## Discussion

This study presents the first detailed analysis of the seasonal activity of *Phlebotomus mascittii*. To the best of our knowledge we report the highest number of trapped individuals of *Ph. mascittii* to date and newly identified bloodmeal hosts of this species.

Even though *Ph. mascittii* is usually reported in very low numbers, it is widely distributed throughout Europe and the predominant sand fly species in Central Europe. Only sporadic findings of other sand fly species, such as *Phlebotomus perniciosus* Newstead, 1911, in Germany [33] or *Phlebotomus simici* Nitzulescu, 1931, in Austria [22], have been reported. In our study, we trapped unexpectedly high numbers of *Ph. mascittii*, which indicates that population densities might be higher than previously reported. Clear differences in trapping numbers were observed between locations and caught specimens per trap-night ranged from 0.2 to 0.9. In Germany, an average of 0.2 caught specimens of *Ph. mascittii* per trap has been reported [34]. Surveys in Southern European countries usually exceed this number by multiple times, as reported from trappings in Southern Italy, where 85 sand flies of various species per trap-night were caught [11].

Higher numbers of collected specimens, namely 184 and 158, were observed in Hummersdorf and Unterpurkla, respectively, where a chicken barn was located on the property, guaranteeing constant host availability, which might lead to increased population densities. In Southern France, trapping success for *Ph. perniciosus* and *Phlebotomus ariasi* Tonnoir, 1921, was strongly associated with host abundance and availability and an extraordinarily high number of sand flies was caught at a poultry barn containing many chickens [35]. Cazan et al. [36] observed *Phlebotomus perfiliewi* Parrot, 1930, to be only present in a chicken shed, but not in other barns at the same property where cattle, horses, pigs and rabbits were kept at a farm in Romania.

Interestingly, the sex ratio was strongly biased towards females ranging from 1/2.4 to 1/16.6. The light traps used in this study are commonly used for trapping phototropic insects including sand flies; however, the effectiveness of this method varies significantly between species and sex [37]. While a shifted sex ratio towards females has been reported for *Ph. mascittii* [3, 34, 35, 38–40], studies on other *Phlebotomus* species often report more captured males than females by light trapping [41, 42]. A male-biased sex ratio among *Lutzomyia longipalpis* Lutz & Neiva, 1912, was found to be associated with higher sand fly densities and more available hosts [43, 44]. However, these factors did not influence the sex ratio in this study when comparing trapping numbers at the four trapping locations. Clearly, trapping methods and ecological factors are associated with the sex ratio of caught specimens. However, activity peaks differed between males and females in this study: male activity peaked in late June and female activity at the end of July. Under laboratory conditions males are also observed to emerge earlier than females and have shorter live spans [45]. This indicates that the observed sex ratios may depend on seasonal activity with this trapping method.

To date, no studies monitoring *Ph. mascittii* activity over a full season have been published and this study presents the first detailed insight into the seasonal dynamics of this species. July and August are the typical months of *Ph. mascittii* activity in Central [4, 6, 34] and Southern European countries [35, 46]. The only exception is a climatically stable tunnel in Corsica, where winter activity of *Ph. mascittii* has been noticed [15]. As we observed *Ph. mascittii* to be highly active by late June in 2018, we adjusted our trapping scheme in 2019, starting regular trappings at the beginning of June to record first activity. A single specimen was again recorded in the first trap night, followed by a week of no activity, indicating that sand fly activity might have recently started. While the length of activity periods varied between locations, a monomodal activity trend was observed at all locations with activity peaks in July at three locations and August in Ratzenau. According to Alten et al. [47] the number of peaks is associated with the number of generations, which suggests a single generation of *Ph. mascittii* in Austria. A monomodal trend was also observed for *Ph. ariasi* in France and *Phlebotomus kandelaki* Shurenkova, 1929, as well as *Phlebotomus balcanicus* Theodor, 1948, in Georgia. Up to three density peaks and substantially longer activity periods are usually observed in countries with lower latitudes such as Portugal, Turkey, Greece or Cyprus [47].

While data on seasonal dynamics of sand flies are available from Mediterranean countries [35, 47, 48], data on activity at the northern boundary of sand fly occurrence are scarce. Recently, Cazan et al. [36] published a study on the seasonal dynamics of *Ph. perfiliewi* in northern Romania approximately at the same latitude as locations surveyed in this study. They observed sand fly activity from July to August, comparably shorter than observed activity periods in our study. Typically mild May and June temperatures in Austria possibly contribute to early sand fly activity starting in early June. Taking constant rising temperatures into account, even earlier sand fly activity might be observed in particularly warm years and in the future; however, this clearly needs further studies.

*Ph. mascittii* was observed to be active at night temperatures as low as 12.8 °C, and first activity was noticed after mean temperatures did not drop below 15 °C and 10 °C minimum. These rather low temperature requirements for activity might contribute to an early start of sand fly activity in June. However, no inference on larval and pupa requirements for development can be drawn from this study; experimental clarification is required. Kasap et al. [14] observed no larval and pupal development of *Phlebotomus papatasi* Scopoli, 1786, at 15 °C under laboratory conditions and a mean temperature of at least 18 °C was necessary for successful rearing. Even though temperature is a driving factor for sand fly activity, similar thresholds for activity of adult sand flies compared to this study were observed for *Ph. ariasi* in France [35] and *Ph. simici* in Austria [22]. In Romania, *Ph. perfiliewi* was not active until minimum temperatures did not fall below 15 °C for 7 days [36].

Despite the low threshold temperature, an association between mean night temperature and sand fly abundance was observed at all locations, which indicates that *Ph. mascittii* actually prefers higher temperatures, but can be active at low temperatures as well. This is in concordance with other surveys, where increasing minimum temperatures were associated with higher trapping success of *Ph. ariasi* in France [35] and *Ph. perfiliewi* in Romania [36]. As *Ph. mascittii* occurs in temperate as well as Mediterranean regions with apparent differences in winter and summer temperatures, it might have a wider temperature tolerance than other sand fly species.

Relative humidity was significantly associated with sand fly abundance, with peak abundance at 80% RH, and decreasing abundances at higher and lower RH. Compared to other species the peak at about 80% is rather high. Significant differences in temperature and humidity requirements were observed between different *Phlebotomus* species on Greek Aegean islands [42]. In contrast to our results for *Ph. mascittii*, the activity of *Ph. perfiliewi* declined with increasing relative humidity in a study from Romania [36].

Interestingly, an increase in barometric pressure was significantly associated with a decrease in sand fly activity, which indicates an active response of *Ph. mascittii* to pressure changes. Tichy et al. [49] experimentally confirmed responses of cockroaches and stick insects to humidity and pressure. The response of sand flies to changes in barometric pressure is widely assumed but data are scarce. In general, a rise in barometric pressure is associated with good weather whereas a drop is associated with poor weather including possible rain; thus, observations in our study are rather unexpected. Herczeg et al. [50] observed an increase of the activity of the hematophagous horse fly (Diptera: Tabanidae) in response to a quickly dropping air pressure prior to storms, concluding that climatic conditions shortly before storms exhibit high humidity and potential blood meal hosts move to shelters and might be more easily available for the horseflies. This might also apply for sand fly activity. Moreover, a weak negative correlation between temperature and air pressure was observed, which might lead to an indirect effect of pressure on sand fly activity through temperature. As we trapped exclusively indoors, this setting might explain a higher tolerance of *Ph. mascittii* to bad weather conditions. This is supported by the absence of a significant association between flight activity and wind speed. As sand flies have a weak flight ability, activity usually decreases with increasing wind speed as observed for various species in Sardinia [51]. However, indoor trapping sites provide shelter for sand flies during bad weather including rainfall and high wind speed. In Spain, trapping sites not exposed to wind showed significantly higher sand fly densities compared to exposed sites [52]. Our data suggest that temperature and humidity can be used as good predictors for sand fly abundance in prospective studies.

The permanent availability of blood host species was shown to have a positive effect on sand fly abundance. Only 5 of a total of 15 collected engorged females could be successfully analyzed by either MALDI-TOF mass spectrometry or DNA sequencing, probably because of late stages of blood meal digestion in the remaining specimens. Degraded DNA makes PCR challenging already 24 h after the blood meal [53], and even though MALDI-TOF peptide mass mapping is possible for longer periods after the blood meal [26], the observed blood bolus in some specimens suggested an advanced stage of digestion, making molecular identification of the blood meal host impossible.

Three engorged females were identified to have fed on chicken. They had been trapped in Unterpurkla and Hummersdorf, where chicken sheds are located at the respective properties. Interestingly, the other two blood meals that could be identified originated from either donkey or horse, although no equid species were present at the respective properties. However, horse barns were located in the range of a few 100 m. Sand flies usually have short flight ranges and only a few have been shown to travel > 1000 m [54]. Altogether, our findings suggest that *Ph. mascittii* is a multi-host feeding species, which is further supported by observations by Grimm et al. [7], who confirmed that one *Ph. mascittii* specimen had fed on human blood. In a recent study, *Leishmania infantum* DNA was detected in an unfed *Ph. mascittii* female in Lower Austria and a dog at the property was observed to be infected with *L. infantum*. The dog most probably had been vertically infected by its mother, which indicates that the sand fly was infected by feeding on the dog [19].

Host availability and identification are not only important for finding new breeding sites and assessing the potential for further dispersal, but also regarding the suspected vector capacity of *Ph. mascittii* for *L. infantum*. Dogs are the main reservoirs for *L. infantum* and they are commonly imported with asymptomatic infections from endemic countries [55]. However, reports of horses infected with *L. infantum* from Germany [56] and with a proposed *Leishmania* species, *Leishmania siamensis*, in Switzerland and Germany indicate that equids might act as reservoir hosts for *Leishmania* species as well [57, 58]. Thus, further clarification of the vector capacity of *Ph. mascittii* and its role in the epidemiology of leishmaniasis in Central Europe is urgently needed.

## Conclusion

Our study presents the first detailed insights into seasonal dynamics and climatic requirements of *Ph. mascittii*. It was shown that population densities are larger than expected and that activity periods are longer than previously reported in Central European countries. Identified blood meals indicate that *Ph. mascittii* feeds on various animals, which could play an important role for potential *Leishmania* transmission. The expected rising temperatures due to the ongoing climate change may increase population densities and elongate activity periods in the future, which could promote further dispersal of the species into new areas. Our study provides valuable data for prospective entomological surveys, which are essential for monitoring changing sand fly populations and to assess the potential spread of *Ph. mascittii* in Austria and Central Europe in general.

## Supplementary Information

**Additional file 1: Table S1.** Number of collected specimens by sex, trap id and month.

**Additional file 2: Table S2.** Sand fly activity period by location and year.

**Additional file 3: Figure S1.** Predictions of sand fly activity and temperature by location.

**Additional file 4: Figure S2.** Predictions of sand fly activity and humidity by location.

**Additional file 5: Figure S3.** Predictions of sand fly activity and air pressure by location.

## Data Availability

All data generated and analysed during this study were included in the article.

## Abbreviations

*coxI*: Cytochrome c oxidase subunit I
MALDI-TOF: Matrix-assisted laser desorption ionization-time of flight
RH: Relative humidity
SD: Standard deviation
Std. error: Standard error
ZIGLMM: Zero-inflated generalized linear mixed model
