# Supplementary material for: Ecology, seasonality and host preferences of Austrian Phlebotomus (Transphlebotomus) mascittii Grassi, 1908, populations
Source: Parasit Vectors. 2021 May 29;14:291. doi: 10.1186/s13071-021-04787-2 (PMC8164323; doi:10.1186/s13071-021-04787-2)
Supplement: Supplementary file 1 — Additional file 1: Table S1. Number of collected specimens by sex, trap id and month. [file 13071_2021_4787_MOESM1_ESM.docx]

**Table S1.** Number of collected specimens by sex, trap id and month.

|  |  |  | **Ro** | |  | **Ra1** | |  | **Ra2** | |  | **Up1** | |  | **Up2** | |  | **Hu1** | |  | **Hu2** | |  | **total** | | |
| --- | --- | --- | --- | --- | --- | --- | --- | --- | --- | --- | --- | --- | --- | --- | --- | --- | --- | --- | --- | --- | --- | --- | --- | --- | --- | --- |
| **month** | **sex** |  | **2018** | **2019** |  | **2018** | **2019** |  | **2018** | **2019** |  | **2018** | **2019** |  | **2018** | **2019** |  | **2018** | **2019** |  | **2018** | **2019** |  | **2018** | **2019** | **total** |
| **June^a^** | **male** |  | 0 | 2 |  | 0 | 0 |  | 0 | 0 |  | 0 | 1 |  | 1 | 1 |  | 4 | 1 |  | 0 | 3 |  | 5 | 8 | 13 |
|  | **female** |  | 1 | 5 |  | 7 | 1 |  | 1 | 1 |  | 1 | 0 |  | 1 | 9 |  | 8 | 1 |  | 2 | 12 |  | 21 | 29 | 50 |
|  | **total** |  | 1 | 7 |  | 7 | 1 |  | 1 | 1 |  | 1 | 1 |  | 2 | 10 |  | 12 | 2 |  | 2 | 15 |  | 26 | 37 | 63 |
| **July** | **male** |  | 3 | 1 |  | 1 | 1 |  | 1 | 0 |  | 2 | 0 |  | 2 | 0 |  | 27 | 1 |  | 7 | 6 |  | 43 | 9 | 52 |
|  | **female** |  | 3 | 12 |  | 23 | 5 |  | 10 | 5 |  | 6 | 30 |  | 42 | 23 |  | 44 | 11 |  | 24 | 15 |  | 152 | 101 | 253 |
|  | **total** |  | 6 | 13 |  | 24 | 6 |  | 11 | 5 |  | 8 | 30 |  | 44 | 23 |  | 71 | 12 |  | 31 | 21 |  | 195 | 110 | 305 |
| **August** | **male** |  | 1 | 2 |  | 1 | 0 |  | 0 | 1 |  | 0 | 0 |  | 2 | 0 |  | 3 | 0 |  | 2 | 0 |  | 9 | 3 | 12 |
|  | **female** |  | 6 | 1 |  | 4 | 5 |  | 3 | 1 |  | 3 | 6 |  | 20 | 8 |  | 3 | 5 |  | 2 | 3 |  | 41 | 29 | 70 |
|  | **total** |  | 7 | 3 |  | 5 | 5 |  | 3 | 2 |  | 3 | 6 |  | 22 | 8 |  | 6 | 5 |  | 4 | 3 |  | 50 | 32 | 82 |
| **September** | **male** |  | 0 | 0 |  | 0 | 0 |  | 0 | 0 |  | 0 | 0 |  | 0 | 0 |  | 0 | 0 |  | 0 | 0 |  | 0 | 0 | 0 |
|  | **female** |  | 0 | 0 |  | 0 | 0 |  | 0 | 0 |  | 0 | 0 |  | 0 | 0 |  | 0 | 0 |  | 0 | 0 |  | 0 | 0 | 0 |
|  | **total** |  | 0 | 0 |  | 0 | 0 |  | 0 | 0 |  | 0 | 0 |  | 0 | 0 |  | 0 | 0 |  | 0 | 0 |  | 0 | 0 | 0 |
| **total** | **male** |  | 4 | 5 |  | 2 | 1 |  | 1 | 1 |  | 2 | 1 |  | 5 | 1 |  | 34 | 2 |  | 9 | 9 |  | 57 | 20 | 77 |
|  | **female** |  | 10 | 18 |  | 34 | 11 |  | 14 | 7 |  | 10 | 36 |  | 63 | 40 |  | 55 | 17 |  | 28 | 30 |  | 214 | 159 | 373 |
|  | **total** |  | 14 | 23 |  | 36 | 12 |  | 15 | 8 |  | 12 | 37 |  | 68 | 41 |  | 89 | 19 |  | 37 | 39 |  | 271 | 179 | 450 |
| ^a^Trapping start on June 28^th^ in 2018. | | | | | | | | | | | | | | | | | | | | | | | | | | |
