# Supplementary material for: Ecology, seasonality and host preferences of Austrian Phlebotomus (Transphlebotomus) mascittii Grassi, 1908, populations
Source: Parasit Vectors. 2021 May 29;14:291. doi: 10.1186/s13071-021-04787-2 (PMC8164323; doi:10.1186/s13071-021-04787-2)
Supplement: Supplementary file 2 — Additional file 2: Table S2. Sand fly activity period by location and year. [file 13071_2021_4787_MOESM2_ESM.docx]

**Table S2.** Sand fly activity period by location and year.

|  |  | **2018** | | | |  | | **2019** | | |  |
| --- | --- | --- | --- | --- | --- | --- | --- | --- | --- | --- | --- |
| **trap** |  | **start^a^** | **stop** | **period (days)** |  | | **start^b^** | | **stop** | **period (days)** | |
| **Ro** |  | 29/06/18 | 24/08/18 | 56 |  | | 13/06/19 | | 28/08/19 | 76 | |
| **Ra** |  | 28/06/18 | 23/08/18 | 56 |  | | 27/06/19 | | 20/08/19 | 54 | |
| **Up** |  | 28/06/18 | 31/08/18 | 64 |  | | 04/06/19 | | 29/08/19 | 86 | |
| **Hu1** |  | 28/06/18 | 31/08/18 | 64 |  | | 19/06/19 | | 23/08/19 | 65 | |
| ^a^trapping started at 28/06/18, ^b^trapping started at 04/06/2019 | | | | | | | | | | |  |
