# Supplementary figures and images for: Ecology, seasonality and host preferences of Austrian Phlebotomus (Transphlebotomus) mascittii Grassi, 1908, populations
Source: Parasit Vectors. 2021 May 29;14:291. doi: 10.1186/s13071-021-04787-2 (PMC8164323; doi:10.1186/s13071-021-04787-2)

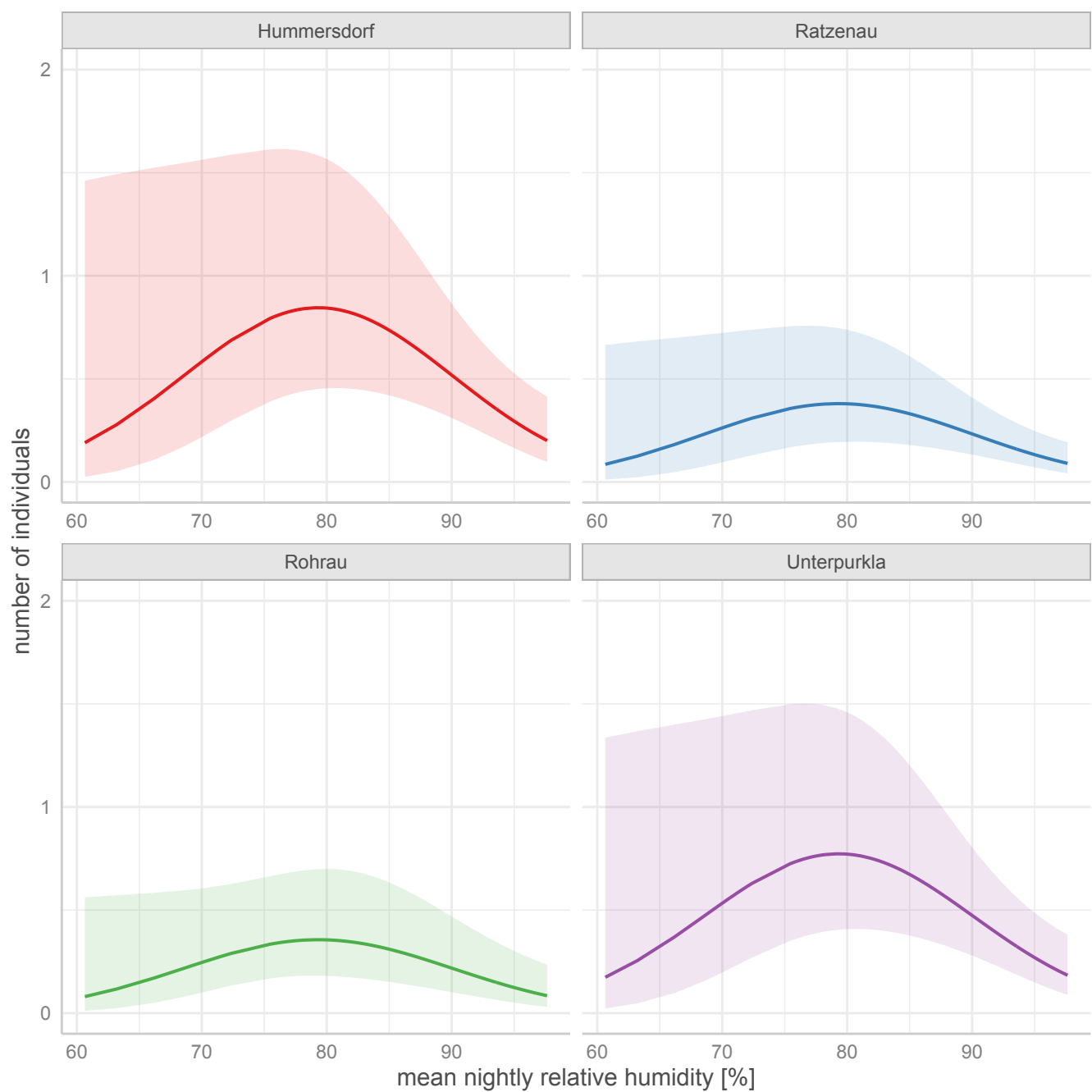

Supplement: Supplementary file 3 — Additional file 3: Figure S1. Predictions of sand fly activity and temperature by location. [file 13071_2021_4787_MOESM3_ESM.pdf]

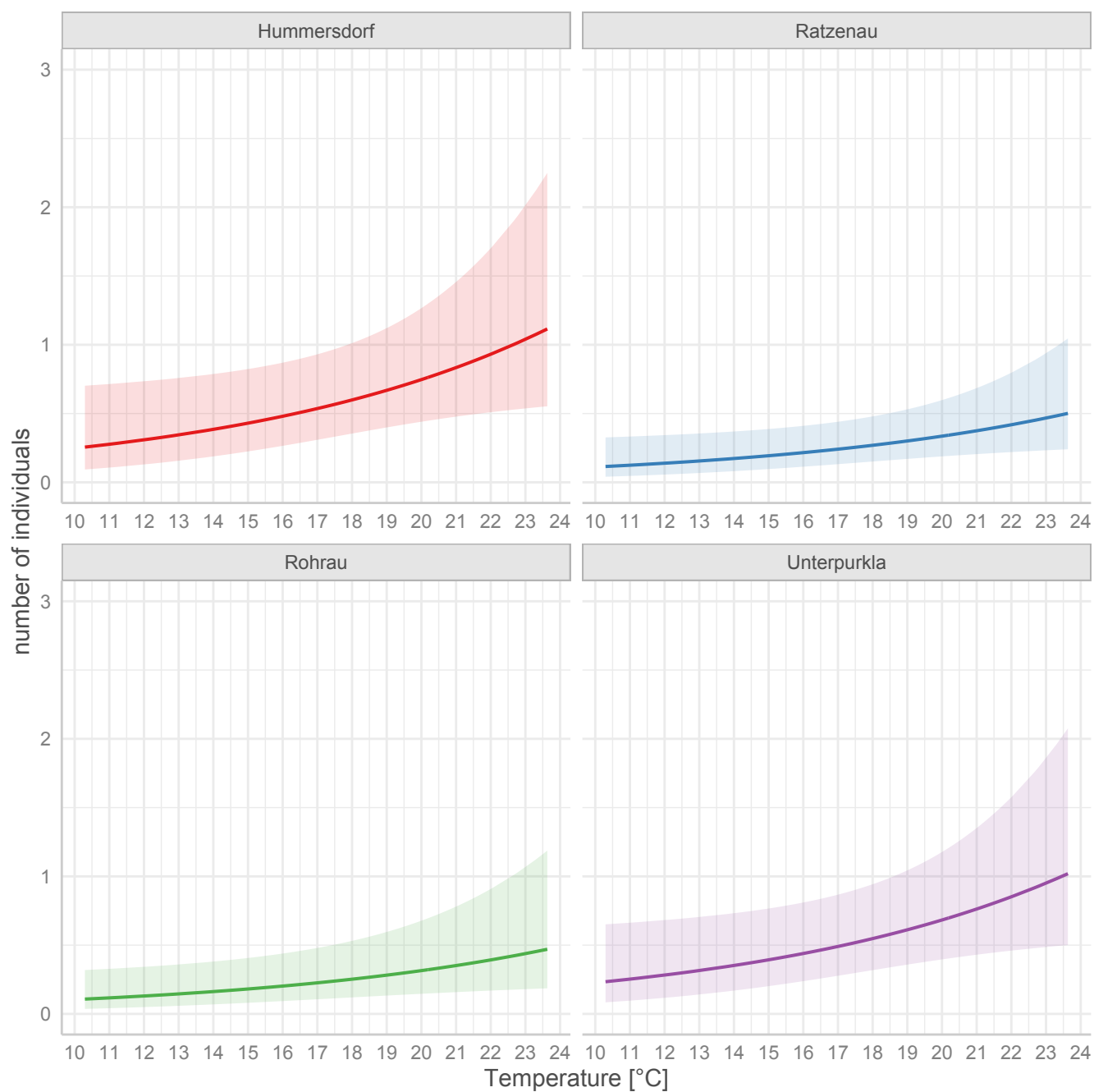

Supplement: Supplementary file 4 — Additional file 4: Figure S2. Predictions of sand fly activity and humidity by location. [file 13071_2021_4787_MOESM4_ESM.pdf]

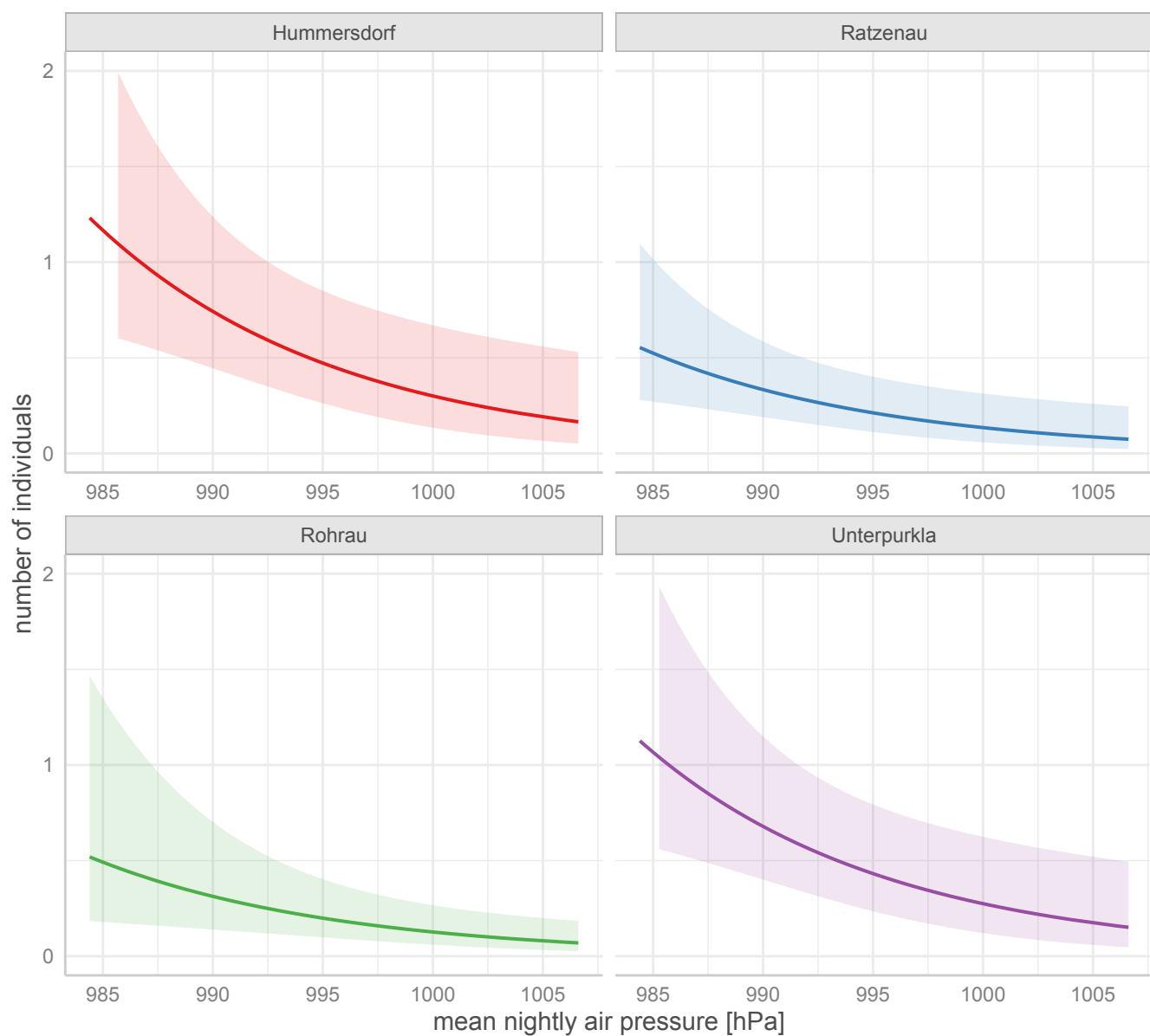

Supplement: Supplementary file 5 — Additional file 5: Figure S3. Predictions of sand fly activity and air pressure by location. [file 13071_2021_4787_MOESM5_ESM.pdf]
